# Ethanol extract of paeonia suffruticosa Andrews (PSE) induced AGS human gastric cancer cell apoptosis via fas-dependent apoptosis and MDM2-p53 pathways

**DOI:** 10.1186/1423-0127-19-82

**Published:** 2012-09-10

**Authors:** Hyeong Sim Choi, Hye-Sook Seo, Ji Hye Kim, Jae-Young Um, Yong Cheol Shin, Seong-Gyu Ko

**Affiliations:** 1 Laboratory of Clinical Biology and Pharmacogenomics and Center for Clinical Research and Genomics, Institute of Oriental Medicine, Kyung Hee University; 2 Department of Pharmacology, Institute of Oriental Medicine, Kyung Hee University; 3 Laboratory of Clinical Biology and Pharmacogenomics, Department of Basic Science of Oriental Medicine, College of Oriental Medicine, Kyung Hee University

**Keywords:** *Paeonia suffruticosa* Andrews, Fas, MDM2, p53, Gastric cancer

## Abstract

**Background:**

The root bark of *Paeonia suffruticosa* Andrews (PSE), also known as Moutan Cortex, has been widely used in Asia to treat various diseases. The molecular mechanisms by which PSE exerts its anti-oxidant and anti-inflammatory activities are well known, but its anti-cancer activity is not yet well understood. Here, we present evidence demonstrating that PSE can be used as a potent anti-cancer agent to treat gastric cancer.

**Methods:**

The effects of the ethanol extract of PSE on cell proliferation were determined using an MTT (1-(4,5-dimethylthiazol-2-yl)-3,5-diphenylformazan) assay. Cell cytotoxicity induced by the PSE extact is measured using an LDH leakage assay. Flow cytometry was used to analyze the cell cycle and to measure the subG0/G1 apoptotic cell fraction. Apoptosis induced by the PSE extact is also examined using a DNA fragmentation assay. Western blot analysis is used to measure the levels of apoptotic proteins such as Fas receptor, caspase-8, caspase-3, PARP, Bax, Bcl-2, MDM2, and p53.

**Results:**

This study demonstrated that treating AGS cells with the PSE extact significantly inhibited cell proliferation and induced cytotoxicity in a dose- and time-dependent manner. The PSE extract also induced apoptosis in AGS cells, as measured by flow cytometry and a DNA fragmentation assay. We found that the PSE extract induced apoptosis via the extrinsic Fas-mediated apoptosis pathway, which was concurrent with the activation of caspases, including caspase-8 and caspase-3, and cleavage of PARP. The MDM2-p53 pathway also played a role in the apoptosis of AGS cells that was induced by the PSE extract.

**Conclusions:**

These results clearly demonstrate that the PSE extact displays growth-suppressive activity and induces apoptosis in AGS cells. Our data suggest that the PSE extact might be a potential anti-cancer agent for gastric cancer.

## Background

Apoptosis, or programmed cell death, occurs under physiological and pharmacological stress in eukaryotic cells [1-4]. The hallmarks of apoptotic cell death include cell shrinkage, membrane blebbing, nuclear fragmentation, and chromatin condensation. These apoptotic changes are due to the proteolytic activity of the caspase proteases [5-7]. Apoptosis is mediated by either extrinsic or intrinsic pathways, and both pathways converge on the activation of effector caspases by initiator caspases. In the extrinsic apoptosis pathway, Fas (also called Apo-1 or CD95) is activated when the cell-surface death receptors are upregulated, and activated Fas can then activate the initiator caspases. The effector caspases are directly cleaved and activated by initiator caspases, resulting in the cleavage, degradation, or activation of other cellular proteins. In the intrinsic apoptosis pathway, mitochondrial outer membrane permeabilization (MOMP) is changed, and the B cell lymphoma 2 (Bcl-2) family plays a central role in cell death regulation. This process leads to the release of pro-apoptotic proteins from the mitochondrial intermembrane space (IMS) [5].

Worldwide, gastric cancer is the second leading cause of cancer-related deaths, with only lung cancer ranking higher [8,9]. The incidence of gastric cancer is high in Eastern Asia, including Korea and Japan, as well as in Eastern Europe, and South America. In contrast, the incidence of gastric cancer is low in North America, Oceania, Northern Europe, Southeast Asia, and Southern Asia [10]. A course of chemotherapy has been shown to be effective in treating gastric cancer, but therapy-associated side effects have also been reported [11]. Although various advances have arisen in gastric cancer management, patient prognosis remains very poor [12]. Therefore, it is essential to find new agents that can be used to enhance the anti-cancer effects of common chemotherapeutic drugs currently being used for gastric cancer treatment [11,12]*.*

Although traditional medicine has been used for thousands of years [13], the molecular mechanisms underlying the effects of these natural products obtained from the extracts of medicinal plants is still unclear [14-16]. Identification of the biologically active compound is also important in traditional medicine [17].

Moutan Cortex, the root bark of *Paeonia suffruticosa* Andrews (PSE), belongs to the Paeoniaceae family [18]. PSE has been widely used in Asia to treat atherosclerosis, infection, inflammation, and cutaneous disease [19]. PSE has been shown to possess potent anti-oxidant, anti-mutagenic, anti-proliferative, anti-invasive [20], anti-arrhythmic [21], anti-inflammation [22], anti-diabetic [23,24], and anti-obesity activities [25]. However, the molecular mechanisms by which PSE exerts its anti-cancer activity are not well understood. In the present study, we investigated whether PSE displays growth suppressive activity and induces apoptosis in AGS cells. Here, we provide evidence that PSE can be used as a potent anti-cancer agent to treat gastric cancer.

## Methods

### Reagents

RPMI 1640, FBS (fetal bovine serum), trypsin-EDTA, and Dulbecco’s phosphate-buffered saline (D-PBS) were purchased from Welgene (Daegu, Korea). The antibiotic-antimycotic used was purchased from Gibco (Grand Island, NY, USA). MTT (1-(4,5-dimethylthiazol-2-yl)-3,5-diphenylformazan; Thiazolyl blue formazan), RNase A, chloroform, potassium acetate, and p53 inhibitor (Pifithrin-α) were purchased from Sigma-Aldrich (St. Louis, MO, USA). An LDH leakage (CytoTox 96® Non-Radioactive Cytotoxicity) assay kit was purchased from Promega (Madison, WI, USA). The pan-caspase inhibitor (z-VAD-*fmk*) was obtained from Calbiochem-Novabiochem (La Jolla, CA, USA). Caspase-8 inhibitor (Z-IETD-*fmk*) and caspase-9 inhibitor (Z-LEHD*-fmk*) were obtained from R&D systems, Inc (Minneapolis, MN, USA). An EZ-western chemiluminescent detection kit was purchased from Daeillab service co. (Seoul, Korea). Cisplatin was obtained from Ildong Pharmaceutical Co., Ltd (Seoul, Korea).

### Antibodies

Fas receptor, procaspase-3, cleaved caspases (−3 and −8), and poly (ADP-ribose) polymerase (PARP) antibodies were purchased from Cell Signaling Technology, Inc (Danvers, MA, USA). Pro- and cleaved caspase-9, Bcl-2, Bax and α-actin antibodies were purchased from Santa Cruz Biotechnology (Santa Cruz, CA, USA). Mouse and rabbit secondary antibodies were from Calbiochem-Novabiochem (La Jolla, CA, USA). Goat secondary antibody was obtained from Jackson ImmunoResearch (West Grove, PA, USA).

### Preparation of *Paeonia suffruticosa* Andrews (PSE) extract

The bark of roots obtained from *Paeonia suffruticosa* Andrews (PSE) used in this research was purchased from Omniherb (Gyeong Buk, Korea). The roots of PSE (100 g) were immersed twice in 1 L of 80% ethanol and then sonicated using an ultra-sonicator (Branson, USA) for 30 min at room temperature. The resulting extract was then filtered through a 0.22 mm filter and concentrated to approximately 100 mL under reducing pressure. The ethanol extracts were dried in a 42°C water bath with the use of a vacuum pump evaporator (Eyela, Japan) and then freeze-dried for 72 h by freeze dryer (Matsushita, Japan). The concentrated extract was then lyophilized, resulting in 20.5 g of powder that was subsequently dissolved in DMSO to prepare a stock solution of 200 mg/mL. The stock solution was stored at −80°C until use.

### Cell culture

AGS (human gastric cancer) cells were generously provided by Dr. H. P. Kim (Seoul National Cancer Institute, Seoul, Korea) and maintained as a monolayer culture in RPMI 1640 medium that was supplemented with 10% FBS and 1% antibiotic-antimycotic at 37°C in a humidified incubator under 5% CO_2_ gas.

### MTT assay

Inhibition of cell proliferation was determined using a MTT assay. Briefly, AGS cells were seeded in a 96 multi-well plate (5x10^3^ cells / 100 μl), and incubated at 37°C. The next day, the cells were treated with the PSE extract (0, 0.01, 0.05, 0.1, 0.25 and 0.5 mg/ml) for 48 or 72 h. After incubation, MTT reagents (0.5 mg/ml) were added to each well, and the plates were incubated in the dark at 37°C for 2 h. The medium was removed, formazan was dissolved in DMSO, and the optical density was measured at 570 nm using an ELISA plate reader. The absorbance correlates with the viability of cells; therefore, the number of cells (% of control) was calculated using the following formula: cell number (% of control) = ((absorbance of cells treated with herb extract) / (absorbance of control cells)) × 100. The control was AGS cells without herb extract treatment.

### LDH leakage assay

Cell cytotoxicity was determined by a LDH leakage assay. Briefly, AGS cells were seeded in a 24 multi-well plate (1.5x10^4^ cells / ml), and incubated at 37°C. The next day, the cells were treated with the PSE extract (0, 0.01, 0.05, 0.1, 0.25 and 0.5 mg/ml) for 48 or 72 h. After incubation, the supernatant was transferred to EP tubes and then centrifuged at 11,000 rpm for 5 min at 4°C. The supernatant was then transferred to new EP tubes. Next, 50 μl of the supernatant was pipetted into 96 multi-well plates, and the supernatant was mixed with 50 μl of CytoTox 96® Non-Radioactive Cytotoxicity assay reagents. After 30 min of incubation in the dark at room temperature, 50 μl of stop solution was added to each well. The absorption values were measured at 492 nm using an ELISA plate reader. The absorbance correlates to the viability of cells; therefore, the number of cells (% of control) was calculated using the following formula: cell number (% of control) = (absorbance of cells treated with herb extract) / (absorbance of control cells) × 100. The control was AGS cells without herb extract treatment.

### Cell proliferation assay

Cells were seeded in 12-well culture plates at a density of 5 × 10^4^ cells/well. The next day, cells were treated with caspase-8 inhibitor or caspase-9 inhibitor in the presence or absence of PSE for 3 days. Cells were harvested by trypsinization, resuspended in 1–2 ml of medium, and counted using a hemocytometer.

### Cell cycle or DNA content analysis by flow cytometry

Cells were seeded in 60 mm dishes (2.5x10^5^ cells/3 ml) and incubated at 37°C. The next day, 200 μg/ml of PSE was directly added to the culture media and incubated for an additional 12, 24, or 36 h. After incubation, the cells were washed with ice-cold PBS, trypsinized, collected in a 15 ml conical tube, and pelleted by centrifugation (1000 x*g*) for 5 min at 4°C. The pellets were washed twice with ice-cold PBS, fixed in 70% ethanol, washed in PBS, resuspended in 300 μl of PBS containing 50 μg/ml propidium iodide (PI) and 50 μg/ml RNase A (Sigma), and incubated in the dark for 15 min at room temperature. The DNA content in each cell nucleus was determined with a FACSCalibur flow cytometer (Becton-Dickinson, San Jose, CA, USA), and the cell cycle was analyzed using ModFit LT V2.0 software.

### DNA fragmentation assay

Cells (6x10^5^ cells/6 ml) were lysed in 500 μl of 0.3 M Tris–HCl (pH 7.5) buffer containing 0.1 M NaCl, 0.01 M EDTA, and 0.2 M sucrose. After vortexing briefly, 25 μl of 10% SDS was added to the samples, and the samples were incubated for 30 min at 37°C. Next, 120 μl of 5 M KOAC (potassium acetate) was added to the samples, and the samples were kept on ice for 1 h. After centrifugation for 10 min at 4°C (at 15,000 rpm for pellet formation), the pellets were removed. The supernatant was treated with 500 μl of TE buffer (10 mM Tris–HCl and 1 mM EDTA, pH 8.0) containing 2 μl of RNase A (10 mg/ml) and was incubated for 30 min at room temperature. An equal volume of phenol/chloroform/isoamyl alcohol (25/24/1; Usb) was added to the supernatant. The supernatant was vortexed gently and centrifuged for 30 min at 4°C (at 15,000 rpm). The supernatant was removed and 400 μl of 100% EtOH and 80 μl of 3 M NaOAC were added to the pellets. The pellets were mixed gently and incubated at −20°C for 30 min. After centrifugation for 30 min at 4°C (at 15,000 rpm), the supernatants were removed. Next, the pellets were washed twice with 500 μl of 70% EtOH and were centrifuged for 30 min at 4°C (at 15,000 rpm). After removal of the supernatant, the pellets were dried and dissolved in 10 μl of TE buffer. The DNA was loaded on a 1.2% agarose gel.

### Western blot analysis

Harvested cells were lysed with buffer containing 20 mM Tris–HCl (pH 7.5), 150 mM NaCl, 1 mM EDTA, 1 mM Na_2_EDTA, 1 mM EGTA, 1% NP-40, 1% sodium deoxycholate, 1 mM Na_3_VO_4_, 1 mM DTT, 1 mM NaF, 1 mM PMSF, and PI cocktail on ice for 30 min. The lysates were cleared by centrifugation at 13,000 rpm for 20 min at 4°C. The supernatant was stored at −70°C until use. The protein concentration was quantified using a Bio-Rad Bradford protein assay (Bio-Rad, Hercules, CA, USA). Next, total proteins (15–20 μg) were electrophoresed using 6-15% reducing SDS-polyacrylamide gels and transferred to nitrocellulose membranes. After blocking with 0.1% Tween-20 in PBS (PBST) containing 1% skim milk and 1% BSA for 1 h, the membranes were incubated overnight at 4°C with the indicated primary antibodies. After washing in 1X PBST for 15 min (3 times x 5 min), the membranes were incubated with diluted enzyme-linked secondary antibodies. After washing in 1X PBST for 1 h (4 times x 15 min), the protein bands were detected using the EZ-western chemiluminescent detection kit and visualized by exposing the membranes to X-ray films. For the studies of caspase-dependent apoptosis, AGS cells were pre-treated with z-VAD-*fmk* (50 μM) for 1 h before treatment with PSE.

### Statistical analysis

All experiments were expressed as the means ± standard deviations (SD) of at least three separate tests. Student’s *t*-test was used for single variable comparisons, and a *p*-value < 0.05 was considered statistically significant.

## Results

### The PSE extract inhibits cell proliferation in AGS cells

**Figure 1:**
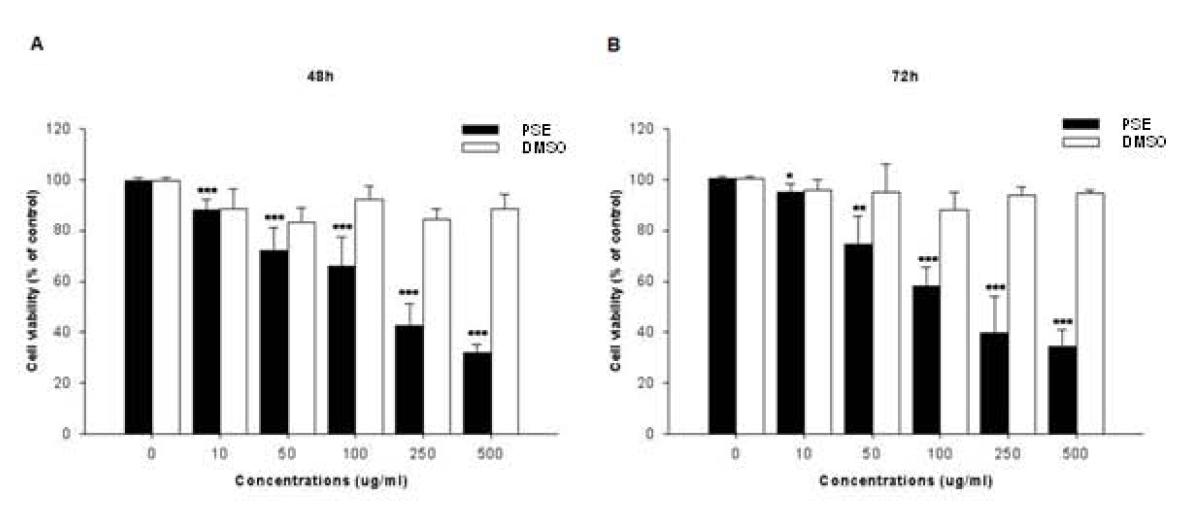
**Effects of PSE on cell viability in AGS gastric cancer cells.** Cell viability was measured using an MTT assay in AGS cells after treatment with multiple concentrations of PSE and DMSO (48 h and 72 h). Cell viability under basal conditions (i.e. no PSE and no DMSO) corresponds to 100%. The data shown are representative of three independent experiments that gave similar results. *Bars,* SD. * *p* < 0.05, **p < 0.01, ***p < 0.001, control versus PSE-treated cells.

To investigate whether the PSE extract inhibits AGS cell growth, the cells were treated with various concentrations (0–500 μg/ml) of the PSE extract for 48 and 72 h, and the viability was measured by a MTT assay. Interestingly, the PSE extract inhibited cell growth in both a dose- and a time-dependent manner (Figure 1). Compared with the control cells, the IC_50_ values of the PSE extract were approximately 220 and 200 μg/ml at 48 and 72 h, respectively. These data suggest that the PSE extract has a clear anti-proliferative effect on AGS cells.

### The PSE extract induces cell cytotoxicity in AGS cells

**Figure 2:**
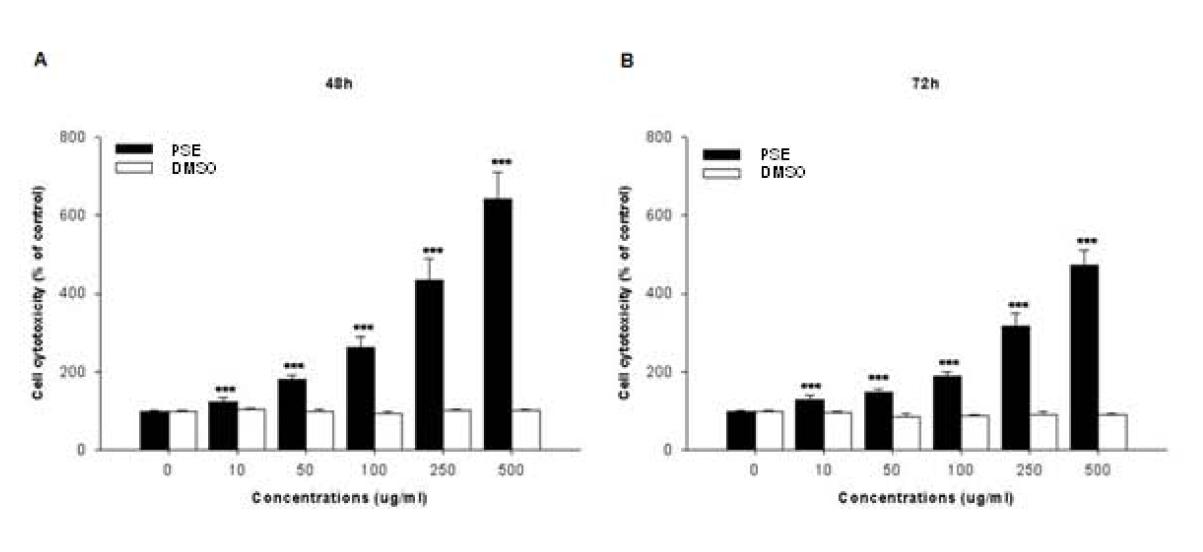
**Effects of PSE on cell cytotoxicity in AGS gastric cancer cells.** Cell cytotoxicity was measured by a LDH leakage assay in AGS cells after treatment with multiple concentrations of PSE and DMSO (48 h and 72 h). Cell cytotoxicity under basal conditions (i.e. no PSE and no DMSO) corresponds to 100 %. The data shown are representative of three independent experiments that gave similar results. *Bars,* SD. ***p < 0.001, control versus PSE-treated cells.

To examine the potential cell cytotoxic effects of the PSE extract in AGS cells, the cells were treated with various concentrations (0–500 μg/ml) of the PSE extract for 48 and 72 h, and the cytotoxicity was measured using CytoTox 96® Non-Radioactive Cytotoxicity assay reagents. We found that PSE killed cells in both a dose- and a time-dependent manner (Figure 2). Compared with the control cells, the LC_50_ values of the PSE extract were approximately 140 and 190 μg/ml at 48 and 72 h, respectively. These results strongly suggest that the anti-proliferative effect of the PSE extract is caused by apoptotic cell death because the PSE extract killed the AGS cells.

### The PSE extract induces apoptosis, apoptotic body formation, and DNA fragmentation in AGS cells

**Figure 3:**
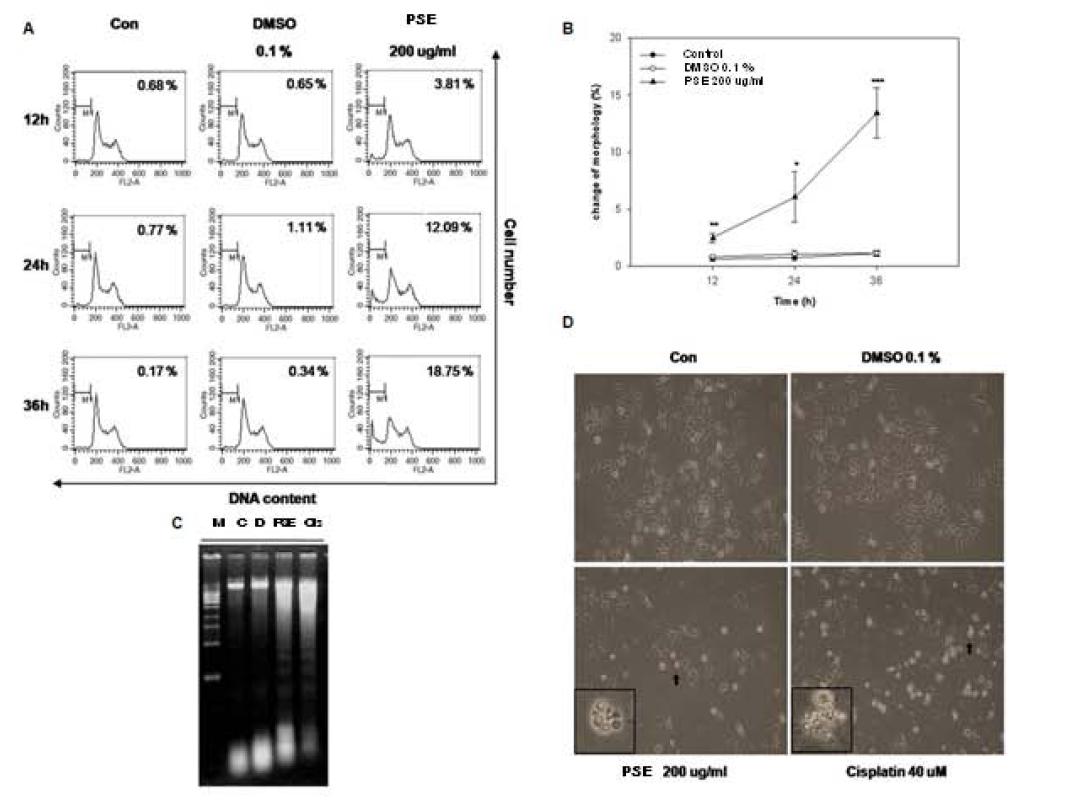
**PSE induced apoptosis, apoptotic body formation, and DNA fragmentation in AGS gastric cancer cells.** (**A**) AGS cells were exposed to 200 μg/ml PSE and 0.1% DMSO for 12 h, 24 h, and 36 h. The DNA content was analyzed using a FACStar flow cytometer, and the percentage of sub-G_1_ phase cells was determined based on a DNA content histogram. (**B**) AGS cells were exposed to 200 μg/ml PSE and 0.1% DMSO for 12 h, 24 h, and 36 h. The percentage of morphologically changed cells was determined based on a FSC/SSC dotplot. (**C**) AGS cells were exposed to 200 μg/ml PSE, 0.1% DMSO and 40 μM cisplatin for 24 h. DNA fragmentation was analyzed using a 1.2% agarose gel for electrophoresis. (**D**) AGS cells were exposed to 200 μg/ml PSE, 0.1% DMSO and 40 μM cisplatin for 24 h. The morphology of cells was imaged using light microscopy (x200). The data shown are representative of three independent experiments that gave similar results. *Bars*, SD. *p < 0.05, **p < 0.01, ***p < 0.001, control versus PSE-treated cells.

To further study the cytotoxic effects of the PSE extract in AGS cells, we treated cells with 200 μg/ml of the PSE extract for 12–36 h and then analyzed the cells for sub-G_1_ DNA contents using flow cytometry. At this concentration, the PSE extract increased the sub-G_1_ apoptotic fractions from 3.81% (12 h) to 18.75% (36 h) in a time-dependent manner. In contrast, neither the untreated control cells nor the DMSO treated cells showed any significant changes in the sub-G_1_ apoptotic fraction (from 0.68% (12 h) to 0.17% (36 h) and from 0.65% (12 h) to 0.34% (36 h), respectively) (Figure 3A).

Morphological analysis demonstrated that AGS cells were divided into either a distinct subpopulation (FSC < 150) or the main population of cells (FSC 150–440) after treatment with the PSE extract (Figure 3B). There was a time-dependent increase in the FSC < 150 population, from 2.47% (12 h) to 13.42% (36 h), after treatment with 200 μg/ml of the PSE extract. In contrast, we did not observe any statistically significant changes in control cells and DMSO treated cells.

A DNA fragmentation ladder showed that the PSE extract and cisplatin (positive control) induced apoptotic DNA ladder formation (Figure 3C). Furthermore, the PSE extract decreased monolayer cell growth and changed cellular morphology (Figure 3D). Cisplatin also showed similar cellular changes. These results confirmed that the PSE extract inhibits the proliferation of AGS cells by inducing apoptosis.

### The PSE extract induces apoptosis via the extrinsic Fas-, caspase-8-, and caspase-3-dependent apoptosis pathway in AGS gastric cancer cells

**Figure 4:**
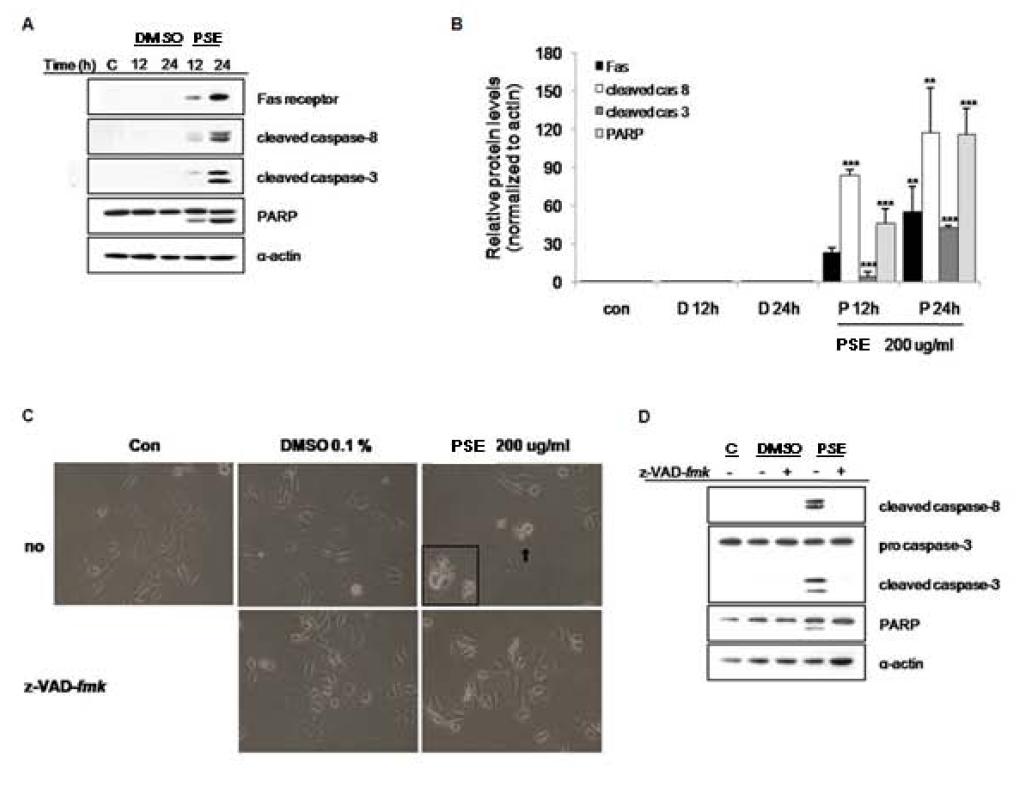
**Fas, caspase-8 and caspase-3-dependent apoptosis is induced by PSE in AGS gastric cancer cells.** (**A**) AGS cells were exposed to 200 μg/ml PSE and 0.1% DMSO for 12 h and 24 h. The cell lysates were separated by SDS-PAGE gel electrophoresis, and Western blotting with specific antibodies was performed (anti-Fas receptor, anti-cleaved caspase-8, anti-cleaved caspase-3, anti-cleaved-PARP, and anti-α-actin). (**B**) The band intensities were normalized to α-actin. (**C**) AGS cells were exposed to 200 μg/ml PSE and 0.1% DMSO either with or without z-VAD-*fmk* (50 □M)*.* The morphology of the cells was imaged using light microscopy (x200). (**D**) AGS cells were exposed to 200 μg/ml PSE and 0.1% DMSO either with or without z-VAD-*fmk* (50 □M) for 12 h, the cell lysates were separated by SDS-PAGE gel electrophoresis, and Western blotting with specific antibodies was performed (anti-procaspase-3, anti-cleaved caspase-8, anti-cleaved caspase-3, anti-cleaved-PARP, and anti-α-actin). The data shown are representative of three independent experiments that gave similar results. *Bars,* SD. **p < 0.01, ***p < 0.001, control versus PSE-treated cells.

We investigated whether the PSE extract induces the extrinsic apoptosis pathway in AGS cells. To address this question, we measured the expression of death receptor signaling-related proteins including Fas, caspase-8, caspase-3, and PARP. We found that the PSE extract activated the Fas death receptor and cleaved caspase-8, caspase-3, and PARP, as seen in Figure 4A and 4B. When we used z-VAD-*fmk* (pan-caspase inhibitor), we found that the growth inhibition induced by the PSE extract was abrogated (Figure 4C). Moreover, the cleavages of caspase-8, caspase-3, and PARP were inhibited by z-VAD-*fmk*, while the PSE extract alone still led to the degradation of caspase-8, caspase-3, and PARP (Figure 4D).Collectively, these results suggest that the apoptotic cell death that is induced by the PSE extract in AGS cells occurs via the caspase-dependent extrinsic apoptosis pathway.

### The PSE extract does not induce apoptosis through the intrinsic mitochondrial apoptosis pathway in AGS cells

**Figure 5:**
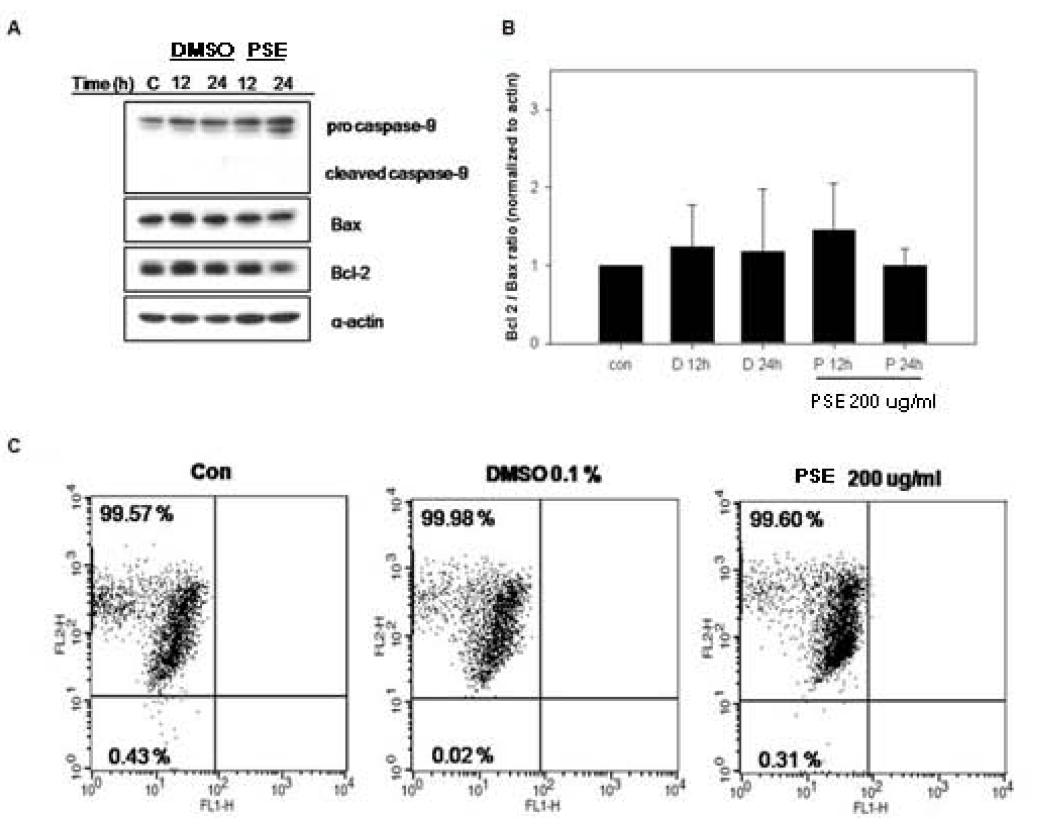
**PSE does not induce the intrinsic apoptosis pathway in AGS gastric cancer cells.** (**A**) AGS cells were exposed to 200 μg/ml PSE and 0.1% DMSO for 12 h and 24 h. The cell lysates were separated by SDS-PAGE gel electrophoresis and Western blotting with specific antibodies was performed (anti-procaspase-9, anti-cleaved caspase-9, anti-cleaved-Bax, anti-cleaved-Bcl-2, and anti-α-actin). (**B**) The quantification of the Bcl-2/Bax ratio of the band density was performed. (**C**) AGS cells were treated with 200 μg/ml PSE and 0.1% DMSO for 24 h, washed twice with PBS and stained with 4 μg/ml JC-1. The data shown are representative of three independent experiments that gave similar results.

Next, we conducted an experiment to determine whether the PSE extract induces the intrinsic mitochondrial apoptosis pathway in AGS cells. We measured the expression of mitochondrial apoptosis pathway-related proteins such as the anti-apoptotic protein Bcl-2 and the pro-apoptotic protein Bax. As seen in Figure 5A and 5B, the expression of Bcl-2 and Bax was not changed by the PSE extract. Moreover, the mitochondrial apoptosis pathway-related caspase, caspase-9, was not cleaved by the PSE extract (Figure 5A).

To confirm the western blot data, we measured the loss of mitochondrial membrane potential (ΔΨm) using JC-1. JC-1 shows a high mitochondrial ΔΨm with intense red fluorescence, forming J-aggregates when the cells are in a healthy non-apoptotic state. In apoptotic or unhealthy cells (especially mitochondria-mediated apoptotic cells), JC-1 shows green fluorescence, forming J-monomers that have a low ΔΨm. The data in Figure 5C demonstrate that the PSE extract did not affect mitochondria membrane potential, as seen by the maintenance of red fluorescence from 100% (control) to 100% (DMSO 0.1%) to 99.91% (PSE). We suggest that the PSE extract does not induce intrinsic mitochondrial apoptosis in AGS cells but induce extrinsic death receptor related apoptosis.

**Figure 6:**
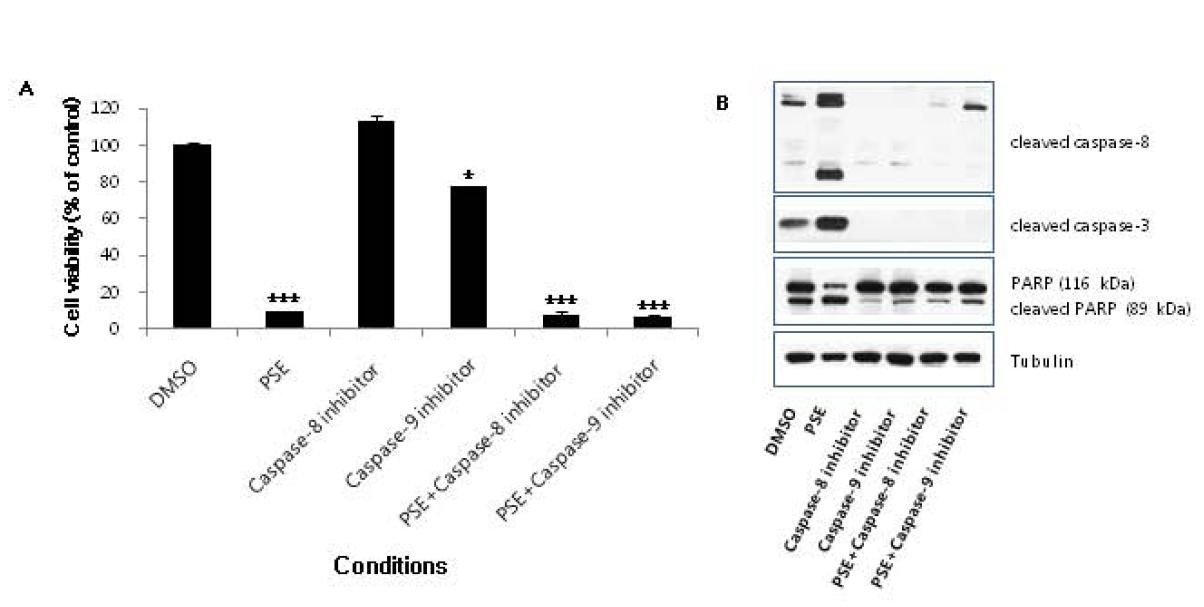
**Effect of caspase-8 inhibitor and caspase-9 inhibitor on the apoptosis induced by PSE in AGS gastric cancer cells.** (**A**) AGS Cells were seeded in 12-well culture plates at a density of 5 × 10^4^ cells/well. The next day, cells were treated with caspase-8 inhibitor (40 □M) or caspase-9 inhibitor (40 □M) in the presence or absence of 200 μg/ml PSE for 3 days. Cells were harvested by trypsinization, resuspended in 1–2 ml of medium, and counted using a hemocytometer. *Bars*, SD. *p < 0.05, ***p < 0.001. (**B**) AGS cells were exposed to 200 μg/ml PSE and 0.1% DMSO either with or without caspase-8 inhibitor (40 □M) and caspase-9 inhibitor (40 □M) for 24 h, the cell lysates were separated by SDS-PAGE gel electrophoresis, and Western blotting with specific antibodies was performed (anti-cleaved caspase-8, anti-cleaved caspase-3, anti-cleaved-PARP, and anti-Tubulin). The data shown are representative of three independent experiments that gave similar results.

To confirm whether the apoptosis induced by the PSE extract is only via extrinsic Fas-, caspase-8-, and caspase-3- dependent pathway, we treated AGS cells with caspase-8 inhibitor (Z-IETD-*fmk*) and caspase-9 inhibitor (Z-LEHD*-fmk*) and performed proliferation assay and western blot. We found that caspase-9 inhibitor alone decreased weakly cell proliferation suggesting that caspase-9 is not key enzyme to induce apoptosis (Figure 6A). We also found that the PSE extract strongly suppressed the cell proliferation even in the presence of caspase-8 inhibitor and caspase-9 inhibitor (Figure 6A). Western blot data demonstrated that cleavages of caspase-8, caspase-3, and PARP were inhibited by caspase-8 inhibitor and caspase-9 inhibitor (Figure 6B). These results confirm that the apoptotic cell death that is induced by the PSE extract in AGS cells occurs via the caspase-dependent extrinsic apoptosis pathway.

### p53-dependent apoptosis is induced by the PSE extract in AGS cells

**Figure 7:**
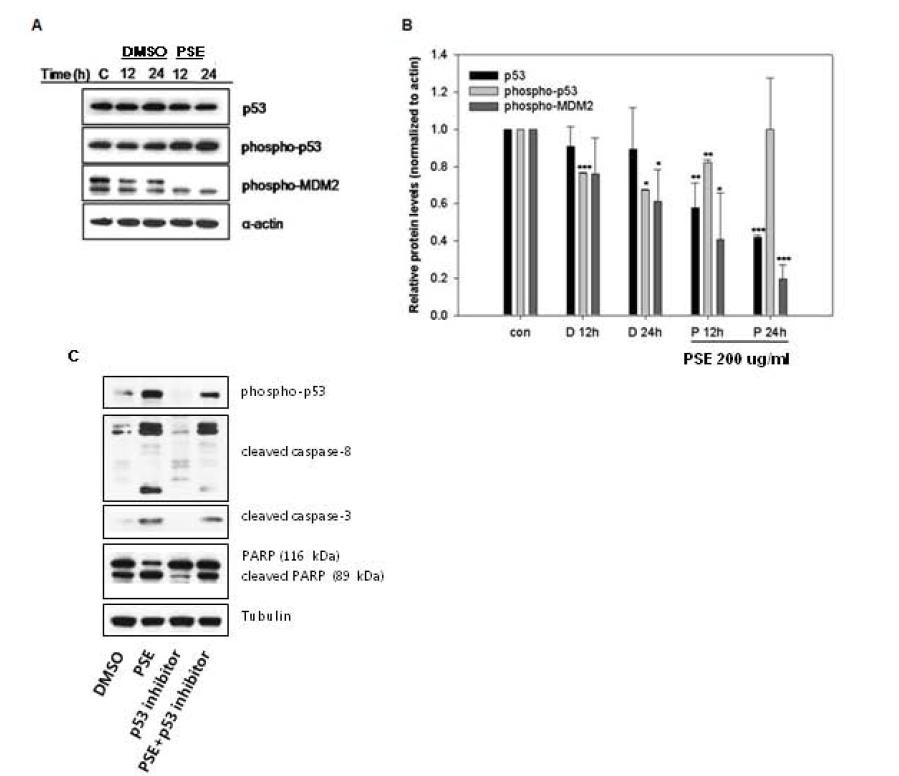
**p53-dependent apoptosis is induced by PSE in AGS gastric cancer cells.** (**A**) AGS cells were exposed to 200 μg/ml PSE and 0.1% DMSO for 12 h and 24 h. The cell lysates were separated by SDS-PAGE gel electrophoresis, and Western blotting with specific antibodies was performed (anti-p53, anti-phospho-p53, anti-phospho-MDM2, and anti-α-actin). (**B**) The band intensities were normalized to α-actin. *Bars,* SD. *p < 0.05, **p < 0.01, ***p < 0.001, control versus PSE-treated cells. (**C**) AGS cells were exposed to 200 μg/ml PSE and 0.1% DMSO for 24 h either with or without p53 inhibitor (Pifithrin-α, 20 μM). The cell lysates were separated by SDS-PAGE gel electrophoresis, and Western blotting with specific antibodies was performed (anti-phospho-p53, anti-cleaved caspase-8, anti-cleaved caspase-3, anti-cleaved-PARP, and anti-Tubulin). The data shown are representative of three independent experiments that gave similar results.

We investigated whether the p53 protein, which plays an important role in the induction of apoptosis, is regulated by the PSE extract in AGS cells. We found that the PSE extract significantly increased the expression of the active form of p53 (phospho-p53), as seen in Figure 7A and 7B. Interestingly, the expression of the active form of MDM2 (phospho-MDM2), a negative regulator of p53, was decreased. To confirm that p53 is implicated in the apoptosis induced by the PSE extract, we treated AGS cells with p53 inhibitor (Pifithrin-α) and performed western blot. We found that cleavages of caspase-8, caspase-3, and PARP were inhibited by p53 inhibitor (Figure 7C). These data suggest that the PSE extract induces apoptosis via the MDM2-p53-dependent pathway in AGS cells.

## Discussion

Although the incidence of gastric cancer has declined worldwide, the death rate continues to rise in Eastern Asian countries [25,26]. In this study, we found that the PSE extract effectively inhibits gastric cancer cell growth via the extrinsic Fas-dependent apoptosis pathway.

Our results demonstrated that the PSE extract suppressed cell growth and induced AGS cell cytotoxicity in a dose- and time-dependent manner. The flow cytometry cell cycle analysis showed that the PSE extract increased the sub-G_1_ apoptotic fraction from 3.81% to 18.75%.

In addition, morphological analysis demonstrated that there was a time-dependent increase in the FSC < 150 population from 2.47% (12 h) to 13.42% (36 h) after treatment with the PSE extract. In addition, the cells treated with either the PSE extract or cisplatin showed morphological changes in the cellular monolayer, including cell shrinkage and membrane blebs, as revealed by microscopic studies [27]. Cisplatin (*cis*-diamminedichloroplatinum(II)) is an anti-cancer agent that is currently used to treat patients with a wide variety of solid tumors, and it acts via a direct interaction with DNA to form DNA adducts [9,28-30]. Cisplatin is known to increase DNA fragmentation, so it was used as a positive control for our study. DNA fragmentation was observed in both the PSE extract - and cisplatin- treated AGS cells, confirming the induction of apoptosis.

Our western blot analyses suggest that the PSE extract induces apoptosis through the activation of the caspase cascade. The present study revealed that treatment with the PSE extract increased Fas receptor expression. Fas (FasL), a cell surface molecule that belongs to the tumor necrosis factor family, binds to its receptor Fas, which leads to apoptosis in Fas-bearing cells [31]. Up-regulated Fas receptor can lead to cleaved caspase-8, caspase-3, and PARP. We observed such Fas-dependent apoptosis signaling in AGS cells treated with the PSE extract. To determine whether the apoptotic process was occurring in a caspase-dependent manner, we pre-treated AGS cells with z-VED-*fmk*, which is a known pan-caspase inhibitor. We found that the growth inhibition induced by the PSE extract was abrogated by z-VAD-*fmk*, and the cleavages of caspase-8, caspase-3, and PARP were inhibited by z-VAD-*fmk*. Therefore, our data demonstrate that the apoptotic process induced by the PSE extract is caspase-dependent. Moreover, we found that caspase-8 inhibitor or caspase-9 inhibitor alone did not strongly affect apoptosis in AGS cells. This event may explain that apoptosis needs the complex action of caspases and caspase does not work alone to affect apoptosis of cancer cells. They need complex mechanism to affect apoptosis. Therefore, caspase-8 or caspase-9 inhibitor alone did not strongly affect apoptosis of AGS gastric cancer cells. PSE seems to trigger complex cascade of caspases action to induce apoptosis. In contrast, the mitochondrial-dependent apoptosis pathway was not activated in AGS cells treated with the PSE extract. Overexpression of Bcl-2, an anti-apoptotic protein, prevents cells from initiating apoptosis, while increase of Bax, a pro-apoptotic protein, induces the intrinsic apoptosis pathway [32]. In the present study, we found that the levels of Bax and Bcl-2 were not changed by the PSE extract. The ratio of Bcl-2/Bax was also unchanged. Furthermore, the PSE extract did not affect mitochondrial membrane potential (MMP), as shown by the maintenance of red fluorescence when the cells were dyed with JC-1. The mitochondrial membrane potential of intact cells could be measured by JC-1, a membrane potential sensitive dye [4]. When the MMP is stable, JC-1 shows red fluorescence, due to the increase of J-aggregate formation. With a loss of MMP, the formation of J-aggregates is reduced, leading to a change from red fluorescence to green fluorescence [30,33]. The lack of change in caspase-9, Bcl-2 and Bax expression that we observed is highly correlated with a stable MMP.

We also describe here that the PSE extract inhibited the proliferation of AGS cells in a p53- and MDM2-dependent manner. After DNA damage, p53 is phosphorylated, which can restrict aberrant cell growth or induce apoptosis [34,35]. Phosphorylation of p53 leads to reduced phosphorylation of MDM2, the negative regulator of p53. MDM2 inactivation is known to be responsible for p53 stabilization (MDM2-p53 pathway) [36,37]. The p53 tumor suppressor inhibits cellular proliferation by inducing apoptosis in response to cellular stresses, including DNA damage, growth factor deprivation, hypoxia, and oncogene activation [38,39]. p53-dependent apoptosis is induced by the caspase proteases and death receptors [40,41] and functions through pro-apoptotic proteins including Bax, Noxa, or PUMA [37].

## Conclusions

**Figure 8:**
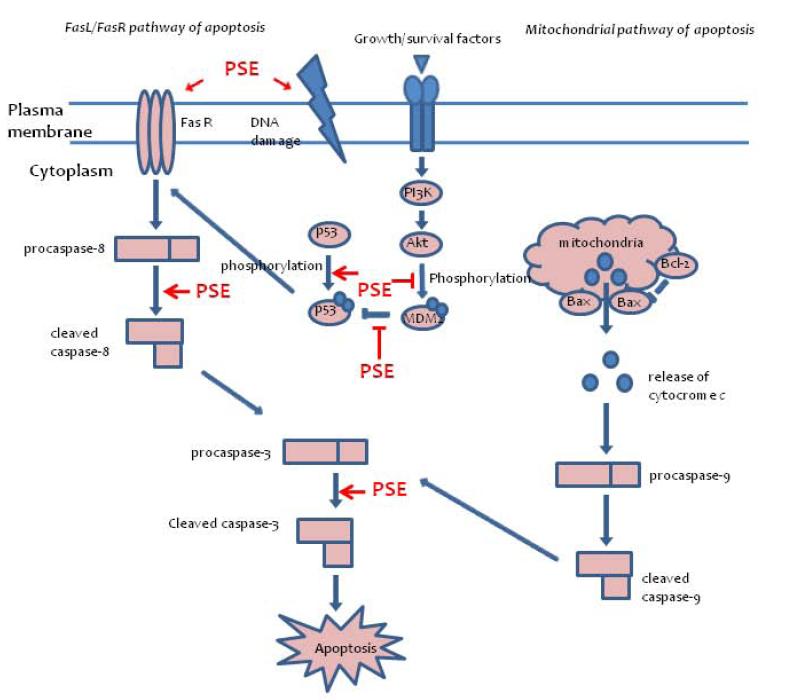
PSE induces p53-dependent and Fas-mediated apoptosis in AGS gastric cancer cells.

In conclusion, this study demonstrated that the PSE extract on AGS cells significantly inhibits cell proliferation and induces apoptotic cell death via the extrinsic Fas-mediated apoptosis pathway (Figure 8). This induction of apoptosis also leads to the activation of caspases such as caspase-8, caspase-3, and cleavage of PARP. The p53-MDM2 pathway also plays a role in the apoptotic cell death of AGS cells that is induced by the PSE extract. Our data clearly demonstrate that the PSE extract might be a potential anticancer agent for gastric cancer.

## Competing interests

The authors declare that they have no competing interests related to this work.

## Authors’ contributions

HSC performed all of the experiments and wrote the manuscript. HSS corrected the English and helped draft the manuscript. JHK, JYU and YCS helped draft the manuscript. SGK critically revised the manuscript for intellectual content and gave final approval of the version to be published.
